# Supercritical CO_2_-Induced Evolution of Alkali-Activated Slag Cements

**DOI:** 10.3390/ma15175873

**Published:** 2022-08-25

**Authors:** Kamasani Chiranjeevi Reddy, Joonho Seo, H. N. Yoon, Seonhyeok Kim, G. M. Kim, H. M. Son, Seunghee Park, Solmoi Park

**Affiliations:** 1 Department of Civil Engineering, Pukyong National University, 45 Yongso-ro, Nam-gu, Busan 48513, Korea; 2 Department of Civil and Environmental Engineering, Korea Advanced Institute of Science and Technology (KAIST), 291 Daehak-ro, Yuseong-gu, Daejeon 34141, Korea; 3 Mineral Processing & Metallurgy Research Center, Resources Utilization Division, Korea Institute of Geoscience and Mineral Resources, 124 Gwahak-ro, Yuseong-gu, Daejeon 34132, Korea; 4 Device Solutions, Samsung Electronics, 1 Samsungjeonja-ro, Hwaseong-si 18448, Korea; 5 School of Civil, Architectural Engineering& Landscape Architecture, Sungkyunkwan University, 2066 Seobu-ro, Jangan-gu, Suwon 16419, Korea

**Keywords:** alkali-activated slag, supercritical CO_2_, carbonation, X-ray diffraction, solid-state NMR

## Abstract

The phase changes in alkali-activated slag samples when exposed to supercritical carbonation were evaluated. Ground granulated blast furnace slag was activated with five different activators. The NaOH, Na_2_SiO_3_, CaO, Na_2_SO_4_, and MgO were used as activators. C-S-H is identified as the main reaction product in all samples along with other minor reaction products. The X-ray diffractograms showed the complete decalcification of C-S-H and the formation of CaCO_3_ polymorphs such as calcite, aragonite, and vaterite. The thermal decomposition of carbonated samples indicates a broader range of CO_2_ decomposition. Formation of highly cross-linked aluminosilicate gel and a reduction in unreacted slag content upon carbonation is observed through ^29^Si and ^27^Al NMR spectroscopy. The observations indicate complete decalcification of C-S-H with formation of highly cross-linked aluminosilicates upon sCO_2_ carbonation. A 20–30% CO_2_ consumption per reacted slag under supercritical conditions is observed.

## 1. Introduction

Portland cement (PC) production is energy intensive and emission intensive; therefore, developing alternate binders with fewer emissions is being explored. Profound investigations are being carried out on alkali-activated slag (AAS) by virtue of its associated low carbon footprint. The AAS binders can produce high mechanical strength, low permeability [1,2,3], and better chemical resistance [4,5,6,7]. The potential of an alternate binder to use as a structural material is comprehensively determined by its mechanical and durability properties. The key role played by carbonation in material degradation accentuates the detailed understanding of carbonation mechanisms. The extent of carbonation in AAS is primarily dependent on the type of activator, CO_2_ concentration, and curing conditions [8,9].

The carbonation rates of alkali-activated slag under service conditions are relatively slow and are comparable to PC [10,11,12]. Several other works have reported higher carbonation in AAS compared to PC as measured from accelerated carbonation tests [13,14,15,16,17,18,19]. Puertas et al. [20] reported a 3- and 10-fold higher carbonation depth in slag activated with NaOH and waterglass, respectively, compared to PC under saturated CO_2_ conditions. While Law et al. [21] reported a 3.5-fold higher carbonation in AAS compared to PC at 20% CO_2_. Bernal et al. [22] observed aggressive carbonation in AAS when exposed to 7% CO_2_. Song et al. [13] reported a decrease in carbonation depths with an increase in Na_2_O content. In another study with 8% Na_2_O, a lower carbonation rate compared to PC was observed by Blim and Atis [14]. A reduction in the carbonation degree was attributed to an associated decrease in porosity and refined pore structure with higher alkalinity [15], while few others have attributed this phenomenon to the enrichment of C-A-S-H with Na^+^, thereby reducing the susceptibility to carbonation [1,23]. Several works have observed the dependence of carbonation rates on the nature of activating solution used in the preparation of alkali-activated slag. Puertas et al. [20] and Ye et al. [24] observed a higher carbonation depth in waterglass activated systems compared to NaOH-activated systems. When slag is activated with sodium silicate coupled with NaOH, the carbonation depths decrease with an increase in Na_2_O content at constant silica modulus [13,14,17]. The degree of carbonation was observed to decrease with an increase in silica modulus at a constant Na_2_O content [15,25]. The presence of MgO has a positive effect on the reduction in the degree of carbonation. With the increase in MgO content, the depth of carbonation has decreased [26,27,28,29]. The presence of MgO has been observed to facilitate a CO_2_ absorption mechanism thereby reducing the carbonation susceptibility of AAS; however, the MgO content in slag is present in fewer quantities.

The carbonation in PC primarily occurs in pore solution and primarily decalcifies the Ca(OH)_2_ into CaCO_3_. The CaCO_3_ thus formed acts as a diffusion barrier inhibiting the further ingress of CO_2_ and carbonation [20]. Alkali-activated slag systems, unlike PC, do not have Ca(OH)_2_ and hence decalcification directly happens in C-S-H [20,30,31]. The disparate carbonation mechanism involved in PC and AAS has exacted the AAS to become more vulnerable to carbonation. This disparity further widens when the samples are subjected to high concentrations of CO_2_ where the degree of carbonation is highly overestimated [15,22,32].

The carbonation of PC when subjected to high concentrations of CO_2_, especially at supercritical concentrations is very rapid [33,34]. The powdered samples subjected to supercritical carbon dioxide were observed to reach their full carbonation within 24 h [35]. The mechanism was reported to be different from accelerated testing. When PC is exposed to flowing supercritical CO_2_ (sCO_2_), the CaCO_3_ is precipitated over the Ca(OH)_2_ crystals limiting its contribution to carbonation and therefore carbonation advances through decalcification of C-S-H [36]. However, in another study, the CaCO_3_ coating is observed to be present in a non-passivating form and hence carbonation is not arrested [37]. The carbonation of Ca(OH)_2_ is observed to form predominantly calcite [36], the formation of different phases is also observed in PC subjected to supercritical CO_2_ [35,38].

The response of PC to sCO_2_ is understood to a certain extent; however, information regarding the structural evolution of AAS exposed to sCO_2_ is highly limited. The studies on carbonation of alkali-activated slag are majorly restricted to sodium hydroxide and sodium silicate-activated slags. An understanding of the effect of carbonation on slag especially under supercritical CO_2_ conditions activated with different types of activators is limited. The structural changes in AAS subjected to carbonation enable us to assess the extent of carbonation and CO_2_ uptake in supercritical conditions. The main focus of the current work is to comprehend the phase changes in AAS systems exposed to sCO_2_. Slag was activated with five different activators and exposed to sCO_2_. The sCO_2_ exposed samples were characterized with XRD, TGA, ^29^Si, and ^27^Al-NMR spectroscopy techniques. The carbonated samples were then compared with uncarbonated samples to assess the phase changes and gel characteristics upon carbonation.

## 2. Experimental Procedure

### 2.1. Materials and Sample Preparation

The X-ray fluorescence chemical composition of the slag (supplied by Chemius Korea, Co., Ltd., Seoul, Korea) used in this study is shown in Table 1. NaOH, Na_2_SiO_3_, Na_2_SO_4_, MgO, and CaO were used as alkali-activators. Alkali-activated slag samples were synthesized at a constant activator dosage of 10 g per 100 g of slag. All samples had an equivalent amount of added water with a powder-to-water ratio of 0.45 based on the dried mass of the materials. The fresh paste samples were poured into a vial, which was placed in a vessel for applying elevated temperature and pressure. The samples were cured in a simulated brine condition to mimic the geologic CO_2_ sequestration conditions by saturating in 1M NaCl solution at 50 °C and an elevated pressure of 10 MPa for 28 days. Note that the samples were removed from the vials to be exposed to brine after 1 day. N_2_ was pumped into the vessel to maintain the pressure during this period. After 28 days the samples were exposed to sCO_2_ by supplying CO_2_ gas into the vessel for another 28 days. The samples were obtained and solvent-exchanged using isopropanol for reaction stoppage before and after exposure to sCO_2_.

### 2.2. Test Methods

The samples were characterized with X-ray diffraction, thermogravimetric analysis, and ^29^Si and ^27^Al magic angle-spinning nuclear magnetic resonance (MAS NMR) spectroscopy. X-ray diffractograms were obtained using an X’Pert Pro X-ray diffractometer (Malvern Panalytical) operating at 30 mA and 40 kV. The powder samples were scanned at a step size of 0.026 °2θ. The thermogravimetric analysis was conducted using a TA Instruments Q600 instrument (PH407) at a heating rate of 10 °C/min in N_2_. Solid-state ^29^Si and ^27^Al MAS NMR experiments were conducted using an Avance III HD instrument (9.4 T, Bruker, Billica, MA, USA) at 79.51 and 104.29 MHz, respectively. A pulse length of 1.6 µs, a spinning rate of 11 kHz, and a repetition delay of 20 s were employed for ^29^Si MAS NMR experiments, while a pulse length of 1.2 µs, a spinning rate of 14 kHz, and a repetition delay of 2 s were employed for ^27^Al MAS NMR experiments. The chemical shifts were referenced to an external TMS and aqueous AlCl_3_ at 0 ppm, respectively. The deconvolution of ^29^Si MAS NMR spectra was conducted using Origin 2020 by introducing Gaussian components at the positions where the relevant sites are known to resonate as reported in previous studies [11,28,39,40,41,42,43,44,45].

## 3. Results

### 3.1. X-ray Diffraction Results

**Figure 1:**
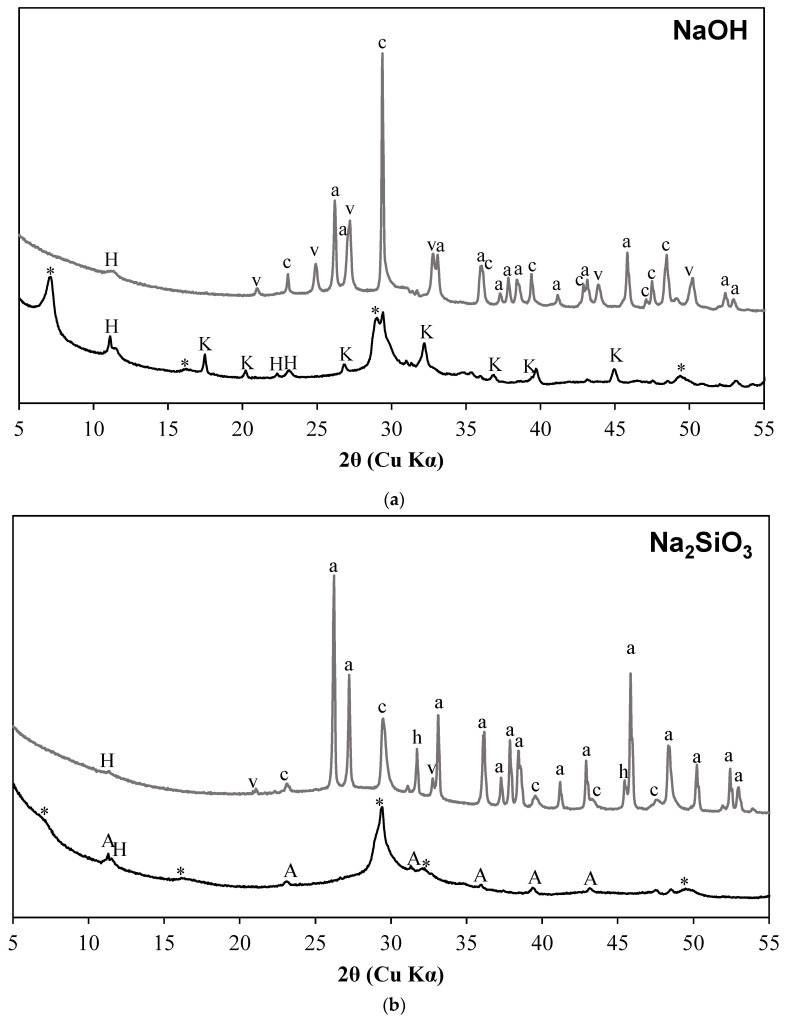

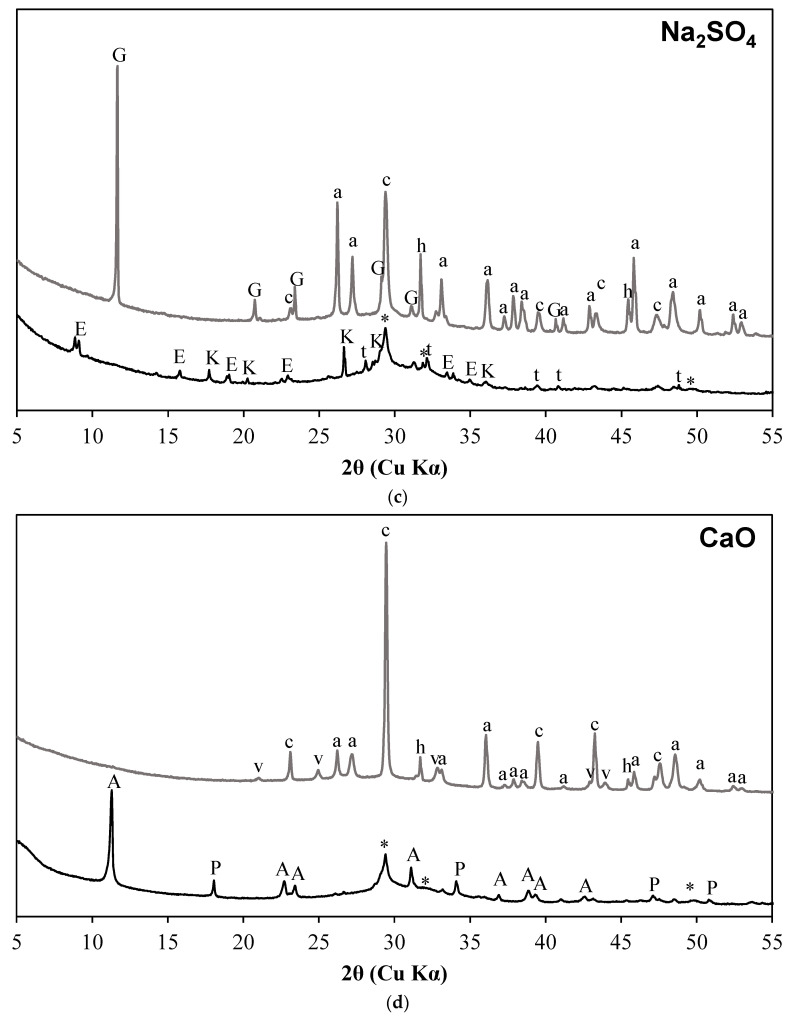

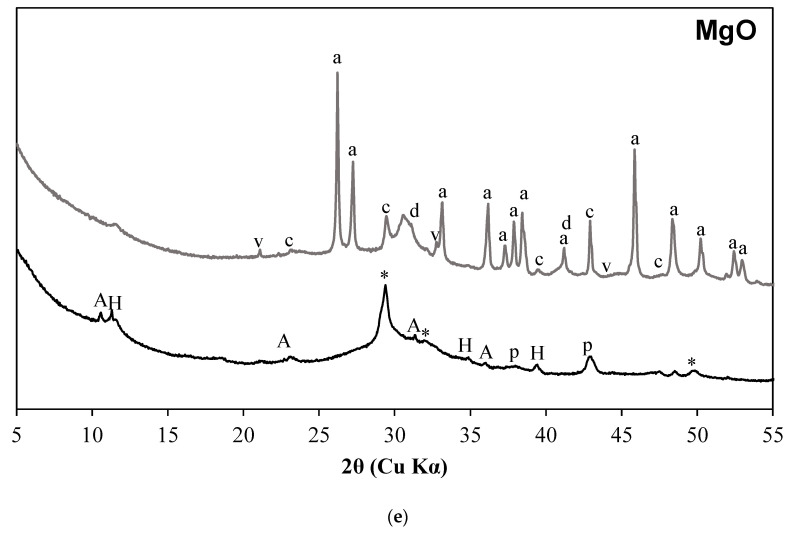
Diffractograms of activated slag before and after being exposed to sCO_2_. (**a**) NaOH (**b**) Na_2_SiO_3_ (**c**) Na_2_SO_4_ (**d**) CaO (**e**) MgO. Samples exposed to sCO_2_ are stacked on top and are represented with a grey color. (*—C-S-H, A—AFm Phases, E—ettringite, G—gypsum, H—hydrotalcite, K—katoite, P—portlandite, S—strätlingite, a—aragonite, c—calcite, h—halite, p—periclase, t—thenardite, v—vaterite, d—dolomite).

The X-ray diffractograms of samples activated with different activating solutions before exposure to sCO_2_ are shown in Figure 1a–e. Each figure contains the diffractograms of the samples before and after being subjected to sCO_2_. The diffractogram of samples before carbonation shows a prominent peak around 29° 2θ irrespective of the activators. The peak at 29° 2θ corresponds to the reflections of poorly crystalline C-S-H with Al incorporation [46,47,48,49,50,51,52]. While the relative position of prominent reflection of C-S-H is similar in all samples prior to carbonation, there are subtle changes in its intensity. The reflection corresponding to C-S-H at approximately 7° 2θ which is prominent in NaOH-activated slag (Figure 1a) has a reduced intensity in Na_2_SiO_3_-activated slag and does not show any intensity in samples activated with other activators. The reduction in the intensity was observed with an increase in Al incorporation into C-S-H [50,53].

The diffractograms of samples activated with NaOH, Na_2_SiO_3_, and MgO consist of peaks corresponding to hydrotalcite, and the presence of katoite was observed in the diffractograms of NaOH- and Na_2_SO_4_-activated samples. In Na_2_SO_4_-activated systems, the phases corresponding to sulfates such as ettringite and thenardite are observed. The presence of sulfates in the activator resulted in the formation of ettringite which is otherwise a rare phase in an alkali-activated slag system [54]. The presence of thenardite indicates the unreacted Na_2_SO_4_ present in the activated system as similarly reported in a previous study [55]. Portlandite reflections are detected in samples activated with CaO. The presence of excess calcium resulted in the precipitation of portlandite in smaller quantities. AFm phases were also formed and are prominently visible in CaO-activated systems, while trace quantities are observed in all other activated systems except the Na_2_SO_4_-activated system.

The samples subjected to sCO_2_ do not exhibit any reflections corresponding to C-S-H. The peaks corresponding to the reflections of calcium carbonate polymorphs were clearly visible in sCO_2_-subjected samples; however, the relative intensities differ in samples activated with different activators. The peaks conforming to hydrotalcite are visible in sCO_2_ samples but of lower intensity compared to that of samples before carbonation. In NaOH-activated slag after sCO_2_ exposure, the calcite reflections are strongly visible followed by almost equivalent vaterite and aragonite forms. The formation of aragonite and calcite are prominently observed along with trace quantities of vaterite in carbonated Na_2_SiO_3_-activated slag. Reflections corresponding to gypsum are visible prominently in carbonated samples of Na_2_SO_4_-activated slag. Carbonation of ettringite in presence of H_2_O is known to result in the formation of gypsum [56,57]. Aragonite is the predominant carbonate phase present in Na_2_SO_4_-activated slag, while the calcite is observed in trace quantities and the vaterite is absent. Calcite is the primary carbonate phase formed in the CaO-activated slag followed by aragonite. Vaterite is present in trace quantities. Aragonite is the only CaCO_3_ polymorph present in the MgO-activated slag. A new peak at approximately 31° 2θ is prominently visible which shows some structural similarities with Ca-dolomite and may have been resulted from the dosage of MgO. Reflections corresponding to halite are present in a few sCO_2_-subjected samples, and that can be attributed to the possible precipitation of halite in some samples in the presence of brine solution employed for curing.

### 3.2. TGA Results

**Figure 2:**
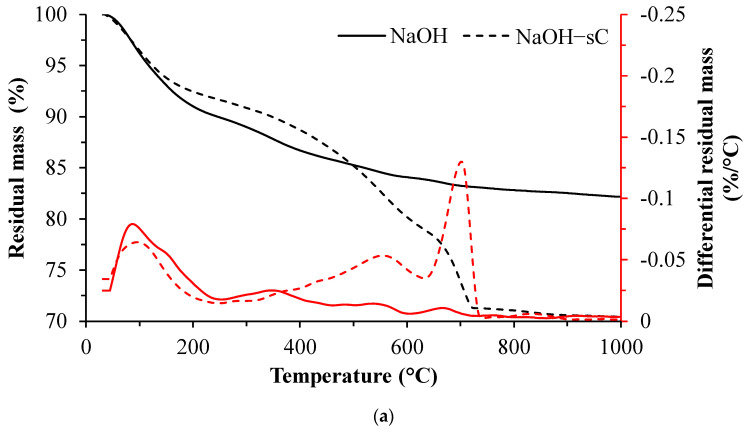

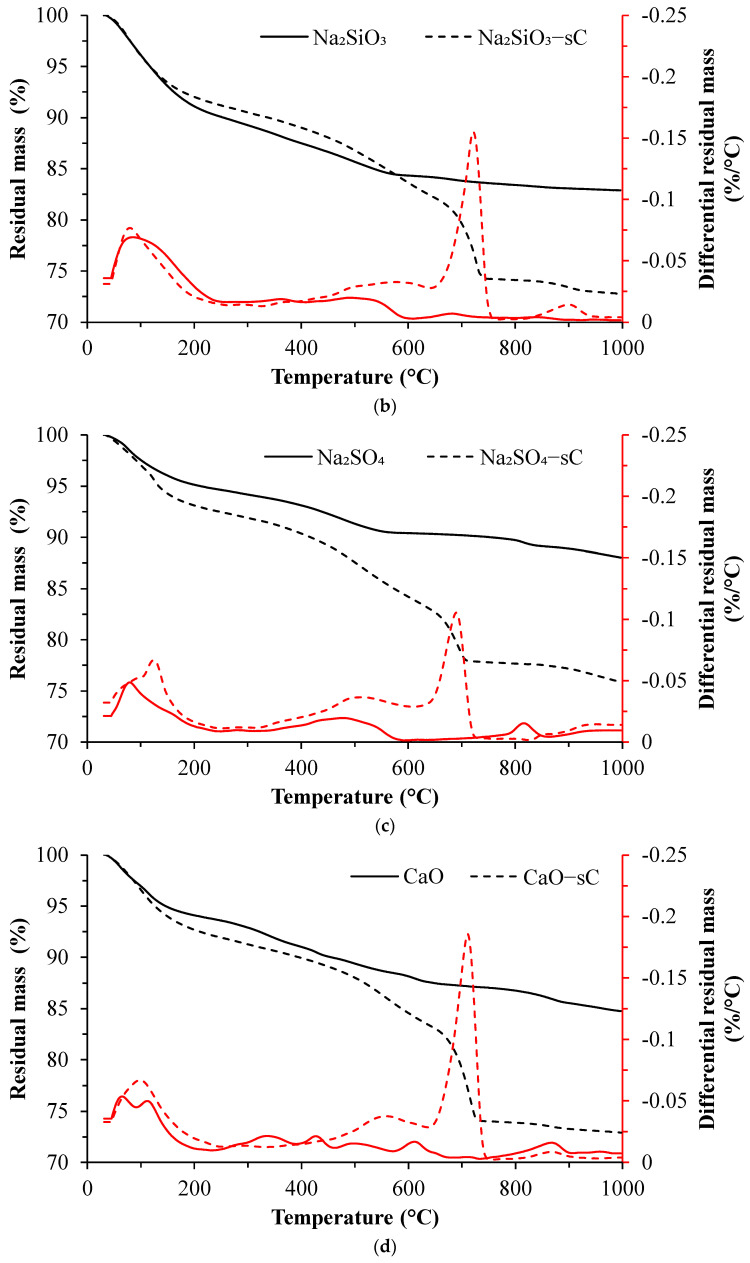

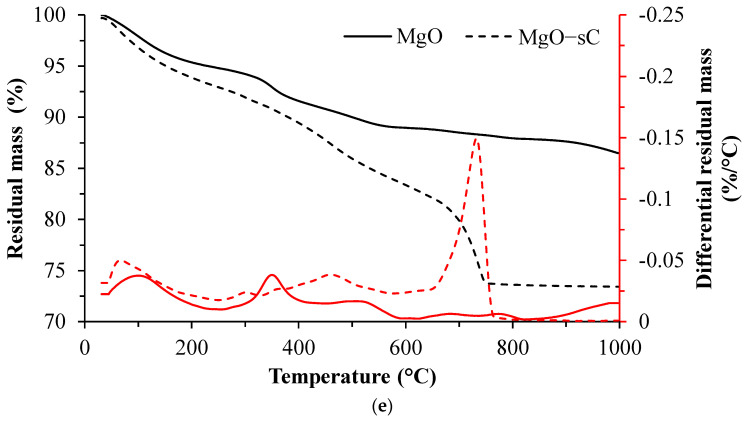
TG/DTG curves of activated slag before and after being exposed to sCO_2_. (**a**) NaOH (**b**) Na_2_SiO_3_ (**c**) Na_2_SO_4_ (**d**) CaO (**e**) MgO. The dotted lines are for the samples exposed to sCO_2_.

The mass loss measurements of slag activated with different activators before and after being subjected to carbonation by sCO_2_ are shown in Figure 2. The differential residual mass is also presented in the same figures. The sample before carbonation has a major mass loss of up to 600 °C due to dehydration of C-S-H along with the minor hydration products. There is a major mass loss due to dehydration from 50 to 200 °C and continued gradual mass loss afterward. The mass loss between 50 to 200 °C is primarily from dehydration of C-S-H. There is no discernable mass loss after 600 °C in uncarbonated samples. The small peak in DTG between 300 and 400 °C in NaOH-activated slag corresponds to the water loss from katoite and hydrotalcite decomposition. Noticeable DTG peaks after 250 °C were not present in Na_2_SiO_3_-activated slag, suggesting no other major phases are present apart from C-S-H. Ettringite present in Na_2_SO_4_-activated samples has a mass loss peak at approximately 100 °C; however, no distinguished peak is present as its mass loss is strongly overlapped with C-S-H. The mass loss peak observed between 300–400 °C in the uncarbonated MgO-activated slag sample is caused by the dihydroxylation and decarbonation of hydrotalcite [58,59].

In the samples after being subjected to sCO_2_, there is a rapid mass loss up to 200 °C; however, the loss is lower than that of the samples before carbonation in NaOH and Na_2_SiO_3_-activated slag. The lower mass loss of up to 200 °C in sCO_2_-subjected samples indicates the higher bound water in hydrates of uncarbonated samples of NaOH and Na_2_SiO_3_. A small mass loss between 500 to 600 °C corresponds to early decarbonation of carbonates in alkali-activated samples [28,45]. The prominent mass loss peak in sCO_2_-subjected samples is visible at 700 °C, which is ascribed to the decomposition of the CaCO_3_. The different polymorphs are converted into calcite when subjected to heating and are subjected to decomposition at similar temperatures [60,61]. The overall mass loss in carbonated samples is 12–15% higher compared to that of uncarbonated samples for any given activator type. In Na_2_SO_4_ samples exposed to sCO_2_, the mass loss peak between 100–150 °C is attributed to the conversion of gypsum to anhydrate via hemihydrate.

### 3.3. ^29^Si MAS NMR

**Figure 3:**
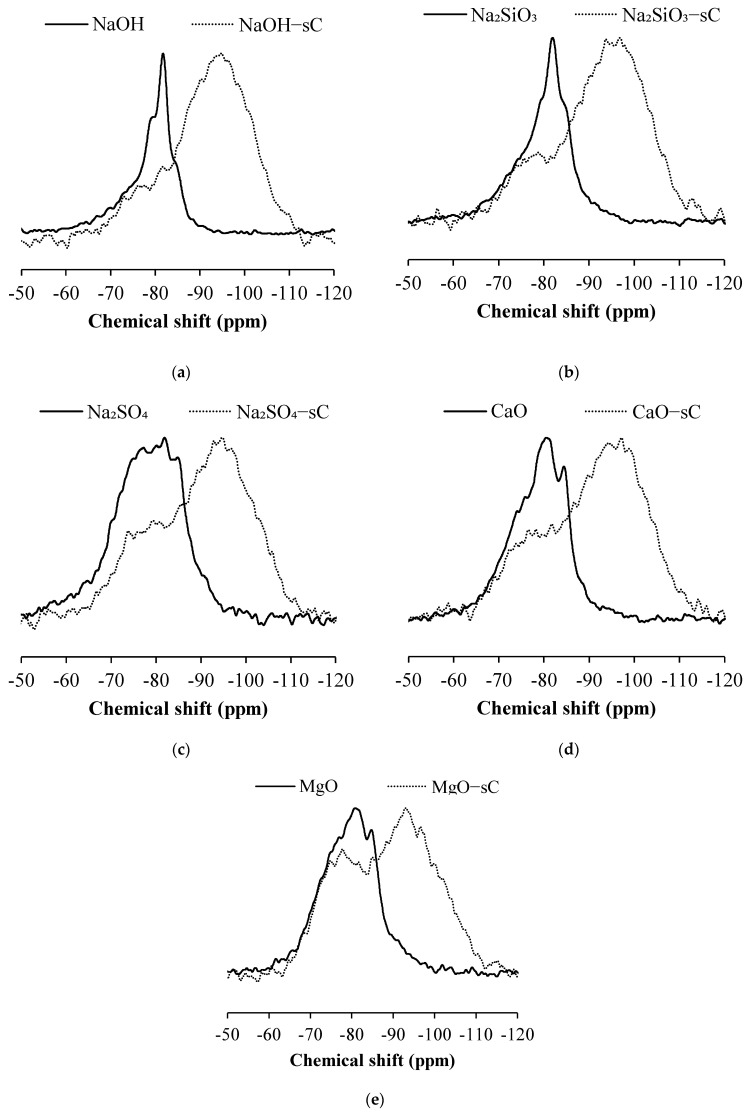
^29^Si MAS NMR spectra of activated slag before and after being exposed to sCO_2_. (**a**) NaOH (**b**) Na_2_SiO_3_ (**c**) Na_2_SO_4_ (**d**) CaO (**e**) MgO. The samples exposed to sCO_2_ are shown in dotted lines.

The ^29^Si MAS NMR spectra of slag activated with different activators are shown in Figure 3. The spectra of carbonated samples are shown in dotted lines. The spectrum of NaOH-activated slag sample showed prominent resonance at −80 and −83 ppm, corresponding to the presence of the Q^1^(II) and Q^2^(1Al) sites. The spectrum also showed smaller resonance peaks at approximately 78 and 86 ppm corresponding to the Q^1^(I) and Q^2^ sites. The Q^1^(I) and Q^1^(II) differ by the charge balancing units which are charge-balanced by monovalent and divalent cations, respectively [43]. The spectrum of uncarbonated sample activated with Na_2_SiO_3_ appear similar to that of NaOH-activated slag and exhibited prominent resonance at −80, −83, and −86 ppm, corresponding to the Q^1^(II), Q^2^(1Al), and Q^2^ sites. The spectrum also consist of smaller resonance peaks at approximately −78 ppm corresponding to the Q^1^(I) site. The spectrum of uncarbonated samples of Na_2_SO_4_-activated sample is more diffused compared to the spectra of the NaOH- and Na_2_SiO_3_-activated system; however, the prominent resonance sites remain at −78, −80, −83, and −86 ppm, corresponding to the Q^1^(I), Q^1^(II), Q^2^(1Al) and Q^2^ sites. The uncarbonated samples activated with CaO and MgO also contain the resonance sites similar to the other systems, but with subtle changes in their intensities.

The prominent resonance has shifted to higher ppm and peaks at approximately –95 ppm in the samples exposed to sCO_2_. The resonance looks more diffused with evenly distributed coordination units. Qualitatively, it is observed that the quantity of lower coordinated bonds has decreased. The resonance at lower chemical shifts is attributed to the presence of decalcified C-A-S-H and the corresponding formation of a highly cross-linked aluminosilicate framework [27,28,45,62]. The relative trends in the resonance spectrum look similar but, there are elusive changes with change in the activator type. While the relative proportion of Q^1^ units is less in NaOH- and Na_2_SiO_3_-activated slag samples exposed to sCO_2_, the MgO-activated slag has significant units followed by CaO- and Na_2_SO_4_-activated systems.

### 3.4. ^27^Al MAS NMR

**Figure 4:**
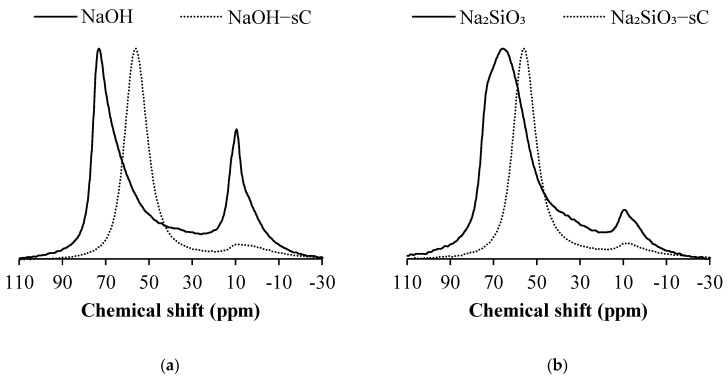

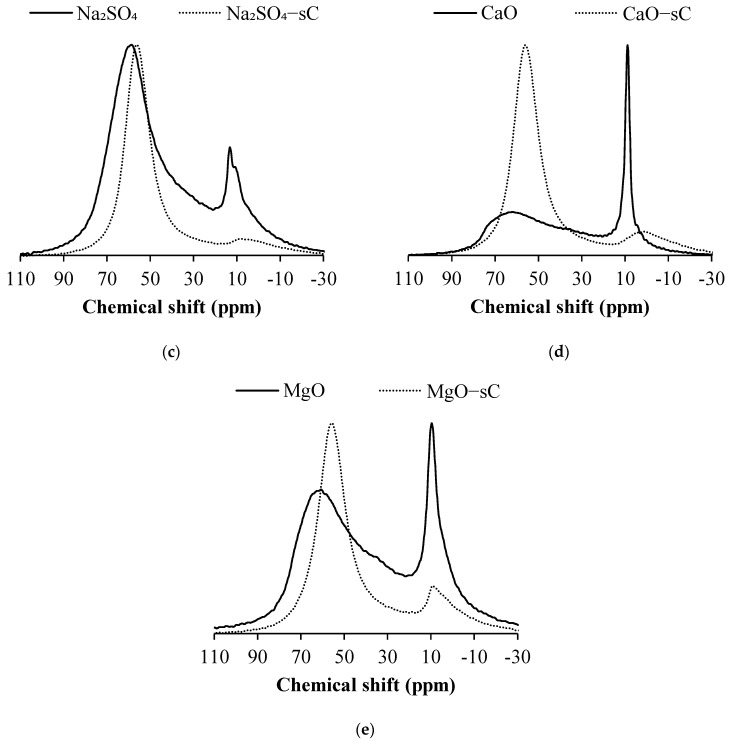
^27^Al MAS NMR spectra of activated slag before and after being exposed to sCO_2_. (**a**) NaOH (**b**) Na_2_SiO_3_ (**c**) Na_2_SO_4_ (**d**) CaO (**e**) MgO. The samples exposed to sCO_2_ are shown in dotted lines.

The ^27^Al NMR spectra of the NaOH-activated samples before and after exposure to sCO_2_ are shown in Figure 4. The samples which are subjected to sCO_2_ are shown in dotted lines. In the samples before carbonation, two prominent resonance zones are observed. The first zone extends between 80 to 50 ppm whereas the second zone spreads in between 20 to –10 ppm with a peak maximum of approximately 10 ppm. In the NaOH-activated slag, the prominent resonance is observed at 73 ppm which corresponds to the q^2^(II) sites of Al, coordinated with tetrahedral Si in C-A-S-H, where Si is charge-balanced with more electropositive divalent cations. The broad tail towards the lower ppm of the first resonance zone indicates the presence of q^3^ sites which resonate at approximately 63 ppm and corresponds to the highly cross-linked Al in C-A-S-H gel. A resonance site at 10 ppm is also observed, which corresponds to the octahedral Al sites present in layered-double hydroxide (LDH, i.e., hydrotalcite). The Na_2_SiO_3_ samples before carbonation exhibit two prominent resonance zones similar to the NaOH samples. The resonance at approximately 70 ppm consists of peaks at 73 and 68 ppm, which correspond to the q^2^(I) and q^2^(II) resonance sites of Al, coordinated with tetrahedral Si in C-A-S-H. The existence of q^3^ sites is indicated by the broad tail towards the lower ppm of the first resonance zone, which peaks at about 63 ppm. The resonance corresponding to the octahedral sites in LDH sites which occurs at 10 ppm is present in uncarbonated samples but is of lower magnitude compared to NaOH-activated samples indicating a reduced amount of hydrotalcite.

Uncarbonated samples of Na_2_SO_4_-activated slag have two prominent resonance zones similar to NaOH and Na_2_SiO_3_. The first resonance site peaks at approximately 60 ppm and consists of overlapped resonance sites at 63 and 58 ppm which corresponds to the q^3^ and q^4^ sites in highly cross-linked aluminosilicates. Resonance events corresponding to the lower coordination units are in small numbers indicating the C-S-H is more cross-linked compared to the NaOH and Na_2_SiO_3_ systems. The resonance at 13 ppm corresponds to the presence of Al in octahedral coordination in ettringite. In CaO-activated slag samples, a resonance site peaks at approximately 60 ppm and consists of overlapped resonance sites at 73, 68, 63, and 58 ppm which corresponds to the q^2^(I), q^2^(II), q^3^, and q^4^ sites aluminosilicates, respectively. The resonance at 8 ppm outweighs the first resonance event and it is attributed to the resonance of Al in AFm phases. The MgO-activated samples also produce resonance at similar sites of other activators; however, the resonance at 10 ppm has larger intensity and is contributed by hydrotalcite and AFm phases.

In all samples of activated slag after being exposed to sCO_2_, the first resonance zone has shifted to lower chemical shifts compared to the corresponding uncarbonated samples. The magnitude of the second resonance zone has decreased drastically upon carbonation. In the carbonated samples, the upfield shift is clearly visible in the first resonance zone and it peaks at 58 ppm. The upfield shift is characterized by more cross-linked aluminosilicates resulting from the decalcification of C-A-S-H. The resonance at 10 ppm diminished substantially in sCO_2_ subjected samples indicating the loss of octahedral sites resulting from the potential carbonation. In NaOH-activated slag, the decrease in resonance at 10 ppm can be attributed to the potential carbonation of hydrotalcite, whereas in Na_2_SiO_3_- and MgO-activated samples, the same has resulted due to the carbonation of hydrotalcite and AFm phases available in trace quantities. In the Na_2_SO_4_-activated system carbonation of ettringite resulted in the loss of octahedral resonance sites. The decrease in the intensity of resonance at 13 ppm in sCO_2_ subjected CaO-activated samples indicates the destabilization of Afm phases and related phase changes upon carbonation.

### 3.5. Discussion

The ^29^Si NMR spectra were deconvoluted using multiple Gaussian peaks and the area under each peak is shown in Table 2. The table also contains the area contributed by the resonance of unreacted slag. The area contributed by the unreacted slag in the uncarbonated Na_2_SO_4_-activated sample is about 75%, which indicates the reaction degree of the slag in Na_2_SO_4_-activated samples is very low. The CaO- and MgO-activated samples consist of ~65% unreacted slag whereas the area of reacted slag has reached about 50% in NaOH- and Na_2_SiO_3_-activated slags [63]. The area contribution from slag has reduced drastically upon carbonation. The amount of unreacted slag in carbonated samples is highest in MgO-activated samples with 28% while NaOH and Na_2_SiO_3_ have a minimal amount of unreacted slag in carbonated samples. The carbonated Na_2_SO_4_- and CaO-activated samples have 21% unreacted slag. The percentage reduction in unreacted slag upon carbonation is least for MgO-activated samples, followed by CaO and Na_2_SO_4_ samples, whereas NaOH and Na_2_SiO_3_ have the highest percentage change in unreacted slag upon carbonation.

The uncarbonated NaOH slag system has a majority of Q^1^(II) sites indicating the formation of C-S-H, while the Na_2_SiO_3_ system has Q^2^(1Al) sites indicating significant Al incorporation into C-S-H. The peak at approximately 7° 2θ in the X-ray diffractogram which is prominently visible in NaOH-activated sample has a reduced intensity in Na_2_SiO_3_ samples. The peak was observed to decrease with an increase in Al/Si ratio in the reaction products [50,53]. The uncarbonated CaO and MgO also have high Q^2^(1Al) units indicating the formation of C-S-H with Al incorporation. The area under Q^3^ and Q^4^ sites with varying Al coordination are very less in uncarbonated samples whereas the corresponding areas in carbonated samples have increased regardless of the activator type. This indicates the formation of highly cross-linked aluminosilicates upon carbonation. The structural changes in C-A-S-H gel to higher cross-linked aluminosilicates are previously observed [24,31,45].

**Figure 5:**
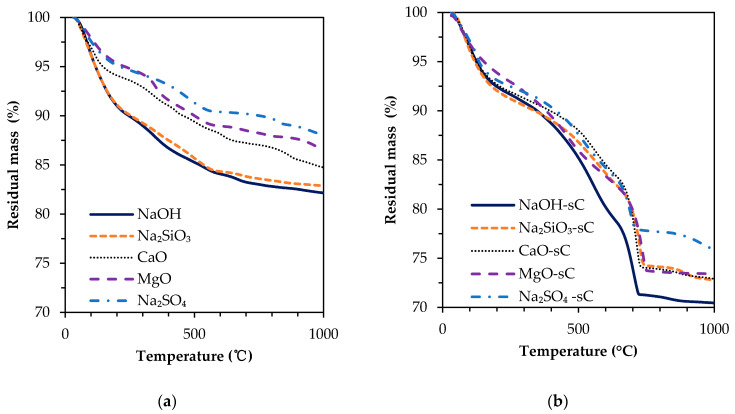
Residual mass curves of alkali-activated slag samples: (**a**) carbonated and (**b**) uncarbonated.

The mass loss curves for all samples are shown in Figure 5. In the uncarbonated samples, the mass loss in NaOH and Na_2_SiO_3_ is very high up to 600 °C whereas Na_2_SO_4_-activated samples have the least mass loss up to 600 °C. The mass loss corresponding to bound water in C-S-H majorly occurs from 50 to 150 °C and a gradual loss up to 600 °C [64]. The mass loss due to katoite, hydrotalcite, and AFM happens within 600 °C [44,65]. The mass loss results up to 600 °C followed an inverse trend as that of the area contributions from unreacted slag in NMR. The NMR results indicate a higher reacted slag content in NaOH and Na_2_SiO_3_ systems. The higher reacted slag results in the formation of larger amounts of C-S-H which holds more water in bound form and hence results in a higher mass loss upon heating up to 600 °C. The reaction product in Na_2_SO_4_ is likely to be in lower quantities owing to the lower extent of slag dissolution and ensued a reduced mass loss. The NMR suggests a similar amount of reacted slag in both CaO- and MgO-activated samples; however, the mass loss in CaO-activated samples is slightly higher, which can be ascribed to the precipitation of portlandite and Afm phases in the presence of excess CaO that is supplied as the activator [66]. The carbonated samples exhibit a similar mass loss up to 400 °C irrespective of the activator type. The water uptake by C-S-H depends on the Ca/Si ratio and increases with Ca/Si ratio. The presence of aluminum also tends to increase water uptake in C-S-H [67]. The Q^1^, Q^2^, and Q^3^ resonance sites in NMR of carbonated samples are nominally equal and the dehydroxylation of hydroxyl ions attached to these sites might have resulted in a similar mass loss up to 400 °C in all carbonated samples. The mass loss beyond 400 °C in carbonated samples is higher in NaOH-activated samples while it is the least for Na_2_SO_4_-activated samples.

The mass of CO_2_ released upon heating in the carbonated samples is estimated by calculating the area of the peak corresponding to the decarbonation under the differential mass loss diagram in Table 3. The tangential approach is employed in area calculations to eliminate the mass contributions due to other phases [68]. The mass of CO_2_ thus calculated was further normalized with the quantity of slag in the carbonated sample. The table shows the normalized mass percentage of CO_2_ released upon heating in carbonated samples. The mass of CO_2_ released per slag upon heating is minimum in Na_2_SO_4_-activated mixes while it is maximum for NaOH samples. The CO_2_ uptake can be better understood when it is compared with reacted slag. Table 3 also shows the mass percentage of CO_2_ normalized to reacted slag quantity. Upon normalization to reacted slag also, the Na_2_SO_4_-activated system has a lower CO_2_ release. The lower extent of slag reaction and preferential precipitation of gypsum limited the amount of calcium available for the formation of calcium carbonates. CaO- and MgO-activated samples have the highest uptake of CO_2_ per reacted slag. The formation of calcium carbonates and magnesium carbonates in presence of Ca- and Mg-based activators, respectively, apart from decalcification of C-S-H have contributed to carbonates [69]. MgO present in the alkali-activated slag was reported to decrease the carbonation [26,27,28,29] whereas when MgO is supplied as an activator resulted in higher contents of CO_2_. NaOH-activated samples have the highest CO_2_ uptake per reacted slag content subsequent to CaO- and MgO-activated systems. The higher degree of reaction in NaOH samples and direct decalcification of C-S-H resulted in higher carbonation [15,70]. Na_2_SiO_3_-activated mixes have an intermediate rate of carbonation with CO_2_ contents. Despite having a similar extent of slag reaction, as indicated from NMR compared to NaOH-activated samples, the Na_2_SiO_3_ samples exhibited a lower carbonation rate. This can be attributed to the lower porosity and dense pore structure in Na_2_SiO_3_-activated systems [15,70].

## 4. Conclusions

The phase transformations and carbonation behavior of alkali-activated slag exposed to sCO_2_ were investigated. The key observations are presented here.

(1) C-S-H with varying degrees of Al incorporation commonly detected in alkali-activated slag systems is observed as the prominent reaction product regardless of the type of activators used in the study.
(2) C-S-H is not observed in the carbonated samples suggesting its complete carbonation. The CaCO_3_ polymorphs such as calcite, aragonite, and vaterite are observed as carbonation products upon exposure to sCO_2_. The relative proportions of different CaCO_3_ polymorphs differed with the change in activator type.
(3) The unreacted slag content decreased drastically with carbonation. About 20–30 g of CO_2_/100 g of reacted slag was consumed for the carbonation of alkali-activated slag samples in sCO_2_ conditions.
(4) The extent of carbonation per reacted slag based on the activator is as follows CaO > MgO > NaOH > Na_2_SiO_3_ > Na_2_SO_4_. The higher C-S-H contents in NaOH-activated slag and the additional carbonation phases resulting from the carbonation of activator in CaO- and MgO-activated slag systems resulted in higher CO_2_ contents.
(5) A decrease in Q^1^, Q^2^ sites and a corresponding increase in the quantity of Q^3^ and Q^4^ units were observed in AAS samples when subjected to sCO_2_, indicating the formation of a highly cross-linked aluminosilicates upon carbonation for all types of activators considered in the study. The formation of highly cross-linked aluminosilicates was also reported previously.

## Figures and Tables

**Table 1:** Chemical composition of blast furnace slag (mass-%).

CaO	SiO_2_	Al_2_O_3_	Fe_2_O_3_	MgO	Na_2_O	K_2_O	SO_3_	TiO_2_	P_2_O_5_	Mn_2_O_3_	SrO	LOI *
43.61	36.18	14.15	0.31	3.46	0.22	0.53	0.37	0.67	0.02	0.37	0.07	0.03

* Loss on ignition, determined in accordance with ASTM C114.

**Table 2:** ^29^Si MAS NMR deconvolution results.

Component	Slag	Q^0^	Q^1^(I)	Q^1^(II)	Q^2^(1Al)	Q^2^	Q^3^(1Al)/Q^4^(4Al)	Q^3^/Q^4^(3Al)	Q^4^(2Al)	Q^4^
Position	−	−74	−78	−80	−83	−86	−89	−93	−100	−107
NaOH	49.4	0.0	1.9	24.1	14.7	8.8	1.1	0.0		
NaOH-sC	10.3	1.7	2.2	3.7	7.8		10.8	26.7	34.9	1.9
Na_2_SiO_3_	49.3	0.0	1.9	6.3	22.7	12.7	4.0	3.2		
Na_2_SiO_3_-sC	7.6	3.3	4.1	3.5		7.2	8.2	20.2	38.4	7.5
Na_2_SO_4_	75.4	0.0	0.4	1.7	4.8	12.7	5.0	0.0		
Na_2_SO_4_-sC	21.5	2.6	0.7	5.0		6.2	7.4	25.2	27.7	3.7
CaO	65.8	0.0	0.6	4.5	17.9	6.9	2.5	1.8		
CaO-sC	20.7	2.0	1.1	3.2		8.0	7.9	15.4	35.4	6.3
MgO	63.8	0.5	0.9	2.7	15.8	8.0	5.4	3.1		
MgO-sC	27.9	3.3	1.3	5.6		9.0	7.1	18.3	21.1	6.4

**Table 3:** CO_2_ uptake of alkali-activated slag samples (g/100 g).

	NaOH	Na_2_SiO_3_	Na_2_SO_4_	CaO	MgO
CO_2_/slag	23.49	18.62	14.88	20.26	17.36
CO_2_/reacted slag	27.99	21.03	21.26	29.99	29.84

## Data Availability

The data presented in this study are available on request from the corresponding author.
